# 2023 OFID Reviewer Recognition List

**DOI:** 10.1093/ofid/ofad646

**Published:** 2023-12-28

The editors of *Open Forum Infectious Diseases* thank the following reviewers for their time and efforts for reviews completed between 1 November 2022 and 31 October 2023. The quality of the journal depends in large part on their expertise.

Justin Aaron

Siti Azrin Ab Hamid

Cybele Abad

Mahsa Abassi

Ume Abbas

Lilian Abbo

Hamad Abdel Hadi

Rima Abdel-Massih

Emily Abdoler

Ahmed Abdul Azim

Syed Muhammad Umer Abdullah

Shira Abeles

George Abraham

Alexandra Abrams-Downey

Chad Achenbach

Hans Ackerman

Carlos Acuña-Villaorduña

Yau Adamu

Alina Adeel

Max Adelman

Florence Ader

Paul Adjei

Brian Agan

Rabia Agha

Wollelaw Agmas

Simon Agolory

Jose Maria Aguado

Daniel Aguilar-Zapata

Sharjeel Ahmad

Faran Ahmad

Mohammad Ahmadi

Abudusalamu Aini

Samuel Aitken

Oscar Bate Akide Ndunge

Ahmed Al Hammadi

Roukaya Al Hammoud

Kalichamy Alagarasu

Abdullah AlAkhras

M. Jahangir Alam

George Alba

salma albahrani

Juan Alberola

John Albin

Owen Albin

Benjamin Albrecht

Bruce Aldred

Sol del Mar Aldrete

Tinsae Alemayehu

Amenah Alghamdi

Majdi Al-Hasan

Vanessa Allen

Tamara Al-maktar

Mohanad Al-Obaidi

Carolyn Alonso

Hanan Al-Turkistani

Richard Alweis

Tanya Amal

Paul Ambrose

K.Rivet Amico

Avnika Amin

Tai Joon An

Melis Anahtar

Åse Bengård Andersen

Tavis Anderson

Daniel Anderson

David Andes

Bruno Andrade

Nick Andrews

Rajesh Aneja

George Anesi

Michael Angarone

Jonathan Angel

Shweta Anjan

Seher Anjum

Joshua Anzinger

Ayesha Appa

Hideki Araoka

Reuben Arasaratnam

Takeshi Arashiro

Ella Ariza-Heredia

Lisa Armitige

Glen Armstrong

Sandra Arnold

David Aronoff

Jenny Aronson

Mansi Arora

Leyla Asadi

Tomefa Asempa

Rachel Asiniwasis

Saima Aslam

William Asquith

Maha Assi

Sabrina Assoumou

Jennifer Audsley

Michael Augenbraun

Paul Auwaerter

Diana Averbuch

Folusakin O. Ayoade

Marwan Azar

Jihoon Baang

Subash Babu

Martin Backer

Oliver Bacon

Abdulla Badawy

Larry Baddour

Melissa Badowski

Shashwatee Bagchi

Natasha Bagdasarian

Jonathan Baghdadi

Nathan Bahr

Francesca Bai

Anthony Bai

Rosemary Bailey

Samuel Bailin

Kristina L. Bajema

Valida Bajrovic

Arthur Baker

Goundappa Balasubramani

Ruchita Balasubramanian

Recep Balik

David Bamberger

David Banach

Ritu Banerjee

Amit Bansal

Aleksandra Barac

Gabriela Barbosa

Amy Barczak

María Dolores Bargues

Jason Barker

Erin Barnes

Joshua Barocas

Jason Barreto

Pennan Barry

Edgar Bautista

Jagadeesh Bayry

Angela Bazzi

Ronald Beaulieu

Roger Bedimo

Edward Belongia

Fatma Ben Abid

Rachel Bender Ignacio

Nicolas Benech

Russell Benefield

Yussef Bennani

Andreas Berge

Catherine Berjohn

Jonathan Berman

Luiz Bermudez

Kyle Bernstein

Nina Le Bert

Mary Bessesen

Hita Bhagat

Ruchi Bhandari

Boudhayan Bhattacharjee

Roby Bhattacharyya

Tanaya Bhowmick

Tihana Bicanic

Cara Bicking Kinsey

Monique Bidell

Urška Bidovec-Stojković

Carlo Bieńkowski

Jose Ramon Blanco

Christopher Bland

Joel Blankson

Lucas Blanton

Anne Blaschke

Mark Bloch

Maxim Bloomfield

Johannes Blum

Peter Boan

Joel Boggan

Isaac Bogoch

Vicente Boix

Sameh Boktor

Sheila Bond

Alexandro Bonifaz

Erin Bonura

P. Brandon Bookstaver

Sophida Boonsathorn

David Boulware

Daniel Bourque

Anna Bowen

Asha Bowen

Anders Boyd

Johannes Boyer

Ben Bradley

Michael Braffman

Daniel Brailita

Carolyn Bramante

Brian Bramson

Angela Branche

Hutton Brandon

Madison Breeden

Meghan Brennan

Jessa Brenon

Carlos Brites

Eeva Broberg

Kristina Brooks

Darron Brown

Elizabeth Brown

Tim A. Bruckner

Dana Bruden

Zabrina Brumme

Giuseppe Bruno

Lou Ann Bruno-Murtha

James Brust

Trond Bruun

Katia Bruxvoort

Ashley Buchanan

Whitney Buckel

Jehan Budak

Jochem B. Buil

Zackery Bulman

Kristen Bunnell

Yvonne Burnett

Jason Burnham

Carey Ann Burnham

Charles Burns

Michael Busch

Fausto Bustos

David Butler

Saira Butt

Morgan Byrne

Jose Cadena

Jorge Calderon Parra

Juan Calix

Jonathon Campbell

Maureen Campion

Anthony Cannella

Sara Cantisan

Jorge Cardenas Alvarez

Yehuda Carmeli

Amy Carr

Jeremy Carr

Dustin Carr

Joseph Carreno

Edgar Carvalho

Agostinho Carvalho

Peggy Carver

Madeline Carwile

Antonella Castagna

Laila Castellino

Natalia Castillo Almeida

Jose Castillo-Mancilla

Kelly Cawcutt

Robert Centor

Jorge Cervantes

Mustafa Cesur

Lelia Chaisson

Jasper Chan

Dominic Chan

Martin Chan

Pranatharthi H Chandrasekar

Sandra Chang

Christina C Chang

Day-Yu Chao

Ryan Chapin

Daniel Chastain

Tulika Chatterjee

Sandip Chavan

Miguel Chavez

Jesse Chavez-van de Hey

Cynthia Chee

Jen-Ting Chen

Jun Chen

Liang Chen

Matthew Cheng

Joseph Cherabie

Supavit Chesdachai

Rusheng Chew

Claire Chewapreecha

Silvia Chiang

Vindana Chibabhai

Brian Chicoine

Ogbonna Chikere

Javier Chinen

John J. Chiosi

Kathleen Chiotos

Lisa Chirch

Witness Chirinda

Cheng-Hsun Chiu

Marc Chong

Yong Pil Chong

Eric Chow

Anthony Chow

Brian Chow

Alexander Chu

Carolyn Chu

Shotaro Chubachi

Jessie Chung

Laura Ciaffi

Christo Cimino

Kimberly C. Claeys

Nina Clark

Andrew Clark

Jonathon Coates

Sally Coburn

Noelle Cocoros

Megan Coffee

Susan Coffin

Stuart Cohen

Steven Cohen

Chari Cohen

Cheryl Cohen

Susan Cohn

Donato Colangelo

Samantha Colledge-Frisby

Jeffrey Collins

Zion Congrave-Wilson

Christopher Conlon

William Connors

German Contreras

Joseph Cooper

Steven Cooperman

Corwin Coppinger

Claudia Cortes

Nicolas Cortes-Penfield

Kimberly Couch

Julien Coussement

Kelsie Cowman

Jill Cowper

Liza Coyer

Brenda Crabtree

Hillary Crandall

John Crane

Duncan Cranendonk

James Crawford

James Crosby

Matthew Crotty

Diego Cruz Vidal

Ashley Cubillos

Marie Culhane

Aubrey Cunnington

Haidee Custodio

James Cutrell

Christopher Czaja

Mary Czech

Sanjeet Dadwal

Laura Damioli

Lynn Damitz

Patrick Danaher

David Dance

Nick Daneman

Keerti Dantuluri

Kusha Davar

Michael David

James Davies

Joshua Davis

Matthew Davis

Mark Dayer

Joao de Almeida Jr

Mark de Boer

Victor De Gruttola

Lorraine Dean

George Deepe

Robert Deiss

Peter DeJonge

Rafael Delgado

Juan Francisco Delgado

Sarah Dellière

Esteban DelPilar

Rachel Denyer

Abhishek Deshpande

Malini DeSilva

Joseph DeSimone

Angela Desmond

Michael DeWitt

Antonio Di Biagio

Zoey Diaz

Kevin Dieckhaus

Daniel Diekema

Tom Dilworth

Daniela DiMarco

Brandon Dionne

Maria Dioverti Prono

Daniel Dirnberger

Olga Djurković-Djaković

Thuy Doan

Sunil Kumar Dodani

Elizabeth Dodds Ashley

Sarah Doernberg

Katherine Doktor

Matthew Dolan

Margaret Doll

Pere Domingo

Valentino D'Onofrio

Fariba Donovan

Gregory Dore

Justin Doritchamou

Nicholas Douglas

Dimitri Drekonja

Philippe Dufresne

Christopher Duggan

Igor Dumic

Ghinwa Dumyati

Michael Dunne

Christine Durand

Kate Dzintars

Ellen Eaton

Joshua Eby

Jessie Edwards

Serin Edwin Erayil

Emily Eichenberger

Mahi Ekambaram

Bassey Ekeng

Zeinab El Boghdadly

Sami El-Dalati

Khalid Eljaaly

Richard Ellison

Marieke Emonts

Gideon Emukule

Rachel Epstein

Guliz Erdem

Nathan Erdmann

Nathan Erdmann

Greg Eschenauer

Zerelda Esquer Garrigos

Vicente Estrada

Benjamin Estrada

Taryn Eubank

Tyler Evans

Christopher Evans

Valeria Fabre

Mark Fajans

Alireza FakhriRavari

Oluwaseun Falade-Nwulia

Julian Falutz

Laura Fanucchi

Esther Fasanmi

Thomas Fekete

Uriel Felsen

Jill Ferdinands

Norma Fernandez

Jose Fernandez-Montero

Victor Ferreira

MU Ferreira

Tristan Ferry

Madiha Fida

Daniel Fierer

Joshua Fierer

Thomas File

Mark Fisher

Christina Fiske

Kathleen Fitch

Charles Flexner

Rommel Flores

Niels Foged

Gary Fong

Emily Ford

Lina Davies Forsman

Johannes Forster

Donald Forthal

Bailey Fosdick

Emily Fox

Denise Marie Francisco

Jeffrey Freiberg

Daniel Freilich

Maristela Freire

Sharon Frey

Stephen Friedman

Daniel Friedman

DeAnna Friedman-Klabanoff

Kohei Fujita

Ayako Fujita

Munehiro Furuichi

Francesco Maria Fusco

Daniel Fuster

Alison Galdys

Maria Galindo

Soren Gantt

Maria Garcia Quesada

Bradley Gardiner

Edward Gardner

Kevin Garey

Ian Gassiep

David Gaston

Timothy Gauthier

Juan Gea-Banacloche

Krista Gens

Jomy George

Dale Gerding

Anthony Gerlach

Erin Gettler

amir ghaffari

Jade GHOSN

Daniele Giacobbe

Thomas Giordano

Marie Gisselsson-Solen

Andreas Glässner

Marshall Glesby

David Glidden

Joseph Glowacki

Casey Godshall

Matthew Goetz

Debra Goff

Shruti Gohil

Sarah Goldberg

Scott Goldsmith

Neal Goldstein

Ellie Goldstein

Delia Goletti

Jonathon Golob

Jesse Goodman

Lindsey Gottlieb

Hetta Gouse

Nelesh Govender

Christopher Graber

Daniel Graciaa

Steve Graham

Robert Greenstein

Christopher Gregory

Eric Gregory

Steven Grinspoon

Andreas Groll

Andreas Groll

Charles Grose

Paolo Antonio Grossi

Daniel Grupel

Huldrych Guenthard

Bruno Guigas

Francisco Guillen-Grima

Yogesh gupta

Asmita Gupte

Pooja Gurram

Sixtine Gurrey

Andrea Guzman

Beth Gyllstrom

James Hadler

Liesl Hagan

Ferry Hagen

Judith Hahn

Ghady Haidar

Andrew Hale

Ronald Hall

Hiroshi Hamada

Fergus Hamilton

Margaret Hammerschlag

Laura Hammitt

Sarah Hammond

Benson M. Hamooya

Andrew Handel

Guy Handley

John Hanna

Rima Hanna-Wakim

Colleen Hanrahan

Marco B. Hansen

Rochelle Hardie

Kristin Harrington

Sadie Harris

Heli Harvala

Tasnim Hasan

Lana Hasan

Mohammad Jahid Hasan

Frederick Hayden

Jillian Hayes

Christopher Hayes

Andrew Haynes

Aniruddha Hazra

Taylor Heald-Sargent

Emily Heil

Jürgen Held

Jannik Helweg-larsen

Vagish Hemmige

Andrés Henao-Martínez

Michael Hendrix

Oryan Henig

Shelbye Herbin

Lauren Nicholas Herrera

Charles Hicks

Lucas Hill

Molly Hillenbrand

Joya-Rita Hindy

Amy Hirsch

Elizabeth Hirsch

Pak-Leung Ho

Nghiem Xuan Hoan

Crystal Hodge

Martin Hoenigl

Leila Hojat

David Holland

Elske Hoornenborg

Thomas Hooton

C. Horsburgh

Leigh Howard

Yu-Hsiang Hsieh

Yin-Chou Hsu

Alice Hsu

Jennifer Hsu

Vanthida Huang

Ying Huang

Jonathan Huggins

Carly Hughes

Ralph Huits

Harry Hull

Te-Yu Hung

Chien-ching Hung

Peter Hunt

Kaisa Huotari

Hermione Hurley

Rocio Hurtado

Shahid Husain

Rezhan Hussein

Benedikt Huttner

Saul Hymes

Kazuki Ide

Elisa Ignatius

Dilek Ince

Nurul Islam

Nahema Issa

Naoya Itoh

Charlotte Jackson

Jesse Jacob

Jana Jacobs

S. Reza Jafarzadeh

Devan Jaganath

Bernadette Jakeman

Sujan Jamarkattel

Suju Jamarkattel

Slobodan M. Janković

Hanna Jarva

Joseph Jarvis

Waleed Javaid

Hana Javaid

Heta Javeri

Javad Javidnia

Raagini Jawa

Meghan Jeffres

Timothy Jenkins

Samuel Jenness

Elizabeth Jenny-Avital

Jorgen Jensen

Conghua Ji

Nick Jilg

Tomasz Jodlowski

Leigh Johnson

Stuart Johnson

Melissa Johnson

Jacob H. Johnson

Christine Johnston

Stephen Jordan

Sandeep Jubbal

Robin Jump

Dima Kabbani

Kamran Kadkhoda

Ron Kagan

E Wangeci Kagucia

Deborah Kahal

Andre Kalil

Lalit Kalra

Ibukunoluwa Kalu

Gautam Kalyatanda

Yiu-Wing Kam

Mala Kaneria

Sheldon Kaplan

Sara Karaba

Andrew Karaba

Styliani Karanika

Adolf Karchmer

Samuel Kariuki

Zahra Kassamali Escobar

shaveta kataria

Louis Katz

Daniel Kaul

Ishminder Kaur

Takeshi Kawaguchi

Michael Kays

Mary Kearney

Caitlin Keighley

Sara Keller

James Kellner

Brian Kendall

Rachel Kenney

Burhan Khan

Saahir Khan

Parisa Khan

Sahil Khanna

Shaghayegh Khojasteh

Celso Khosa

April Killikelly

Arthur Kim

Andy Kim

Do Young Kim

Simeon Kimmel

Muneyoshi Kimura

Travis King

Ben King

Madeline King-Patel

Satoshi Kitaura

Ellen Kitchell

Quratulain Kizilbash

Kendall Kling

Marjolein Knoester

Young Jin Ko

Wei Li Koay

John Koethe

Alan Koff

Maheswara Reddy Koppula

Krishna Kiran Kota

Douglas Krakower

Anna Kramvis

Molly B. Kraus

Peter Krause

John Kubale

James Kublin

Wesley Kufel

Samir Kumar-Singh

Nitin Das Kunnathu Puthanveedu

Ashlan Kunz Coyne

Krutika Kuppalli

Joseph Kuti

Nakwon Kwak

Kathryn Kyler

Ricardo La Hoz

Diana lacob

Oliver Laeyendecker

Botond Lakatos

John Lam

Jennifer Lam

Patrick Lammie

Harry Lampiris

Yinghua Lan

Bradley Langford

Fanny Lanternier

Paul Lantos

Cristina Lanzas

Romaric Larcher

Jeff Larnard

Matthew Laurens

Luca Laurenti

Susana Lazarte

David Lebeaux

Shirley Lecher

Julia Lechuga

Francesca Lee

Yeon Joo Lee

Matthew Lee

Todd Lee

Keun Lee

Tida Lee

Jacob Lemieux

Scott Letendre

Alyssa Letourneau

Donald Levine

Russell Lewis

Joseph Lewis

Yijia Li

You Li

Liang Li

Jonathan Li

Patrick Lillie

Benjamin Linas

Beth Linas

Michail Lionakis

Jessica Little

Catherine Liu

Carson Lo

Nathan Lo

Thomas Lodise

Chris Longenecker

Angela Loo

Bruno Ali Lopez Luis

Olivier Lortholary

Janice Louie

Kym Lowry

Grace Lui

Daniel Lupu

Vera Luther

John Lynch

Conan MacDougall

Shabir Madhi

Joanne Maffei

Christina Maguire

Allison Mah

Monica Mahoney

Willem Mak

Gannon Mak

Rita Makabayi-Mugabe

Vincenzo Malagnino

Alexandre Malek

Prashant Malhotra

Maricar Malinis

Ashwini Mallad

Gervillien Malonga

Carlos Malvestutto

Denis Malvy

Kiran K. Mangalaparthi

Kalpana Manthiram

Peter Maple

Julio Maquera-Afaray

Alberto Enrico Maraolo

Pedro Henrique Ferreira Marçal

Jasmine Marcelin

Arnaud Marchant

Vince Marconi

Luis Marcos

Joseph Marcus

Laura Marks

Kristen Marks

Adriana Marques

Theodore Marras

Gaetano Marrone

Terry Marryshow

Thomas Martin

Esteban Martinez

Elisa Martro

Mark Marzinke

Santiago Mas-Coma

Carmen Masson

Wilfredo Matias

Yasser Matran

Philippa Matthews

Ryan Maves

celia maxwell

Nadia Mazarakis

Patrick Mazi

James McCarthy

Todd McCarty

Kevin W McConeghy

David McConnell

Margaret McCort

Erin McCreary

Donna Hubbard McCree

Emily McDonald

Ian McGovern

Sarah McGuinness

James McKinnell

David McKinsey

Angela McLaughlin

Huong McLean

Jonathon McNeil

Carla McWilliams

Paul S. Mead

Richard Medford

Ashley Meehan

Nirja Mehta

Sanjay Mehta

Aneesh Mehta

James Meiring

Jessica Meisner

Carlos Mejia-Chew

Michael Melia

Alexandra Mellis

Nickoo Merati

Marco Merli

David Meya

Eric Meyerowitz

Sylvain Meylan

Héctor David Meza-Comparán

Katerina Meznikova

Marisa Miceli

Kathryn Miele

Nkuchia M'ikanatha

Jovana Milic

Loren Miller

Molly Miller

Lesley Miller

Aaron Miller

Emily Miller

John Mills

Zaw Min

Daniel Minter

Muhammad Shah Miran

Victor Daniel Miron

Elizabeth Misch

Yusuke Miyazato

Juliet Mmerem

Ryan Moenster

Amir Mohareb

Kyle Molina

Paul Monach

Lea Monday

Brian Montague

Martha Montgomery

Erica Moodie

Ganga Moorthy

Konosuke Morimoto

Taylor Morrisette

Charalampos Moschopoulos

Emily Mosites

Heba Mostafa

Babatunde Motayo

Jianbing Mu

Philip Mudd

Nicolas Mueller

Emmanuel Mugisha

Victor Mulanovich

Sonal Munsiff

Richard Murphy

Jason Mwenda

Edison Mworozi

Ryan Mynatt

Irving Nachamkin

Melanie Nadeau

Khalil Nadim

Shivdas Naik

Yu Nakagama

Hannah Nam

Trusha Nana

Neilanjan Nandi

Shivakumar Narayanan

Masashi Narita

Anne Neilan

Jeremy Nel

Saman Nematollahi

Dionysios Neofytos

Lior Nesher

Valerie Ng

Thundon Ngamprasertchai

Julia Nguyen

Ank Nijhawan

Ran Nir-Paz

Hera Nirwati

Obi Nnedu

Sebastian Noe

David Nokes

Nathanial Nolan

Johan Nordgren

Priya Nori

Brianna Norton

Farzad Noubary

Richard Novak

Robertus Nugroho

Marina Núñez

Andrew Nunn

Karam Obeid

Bright Ocansey

Elise O'Connell

Camila Odio

Nicholas O'Donnell

Michael Okaikoi

Nwora Okeke

Tudor Rares Olariu

Ioana Diana Olaru

Alessandra Oliva

Loice Ombajo

Eloy Ordaya

Jose Orejas

Verner Orish

Chloe Orkin

Nina Orsini

Jesse O'Shea

Kengo Oshima

Joshua Osowicki

Belinda Ostrowsky

Kanako Otani

Caitlin Otto

Kevin Outterson

Edgar Overton

Kimberly Page

Amit Pai

Federico Palacio-Bedoya

A. David Paltiel

Paul Palumbo

Evelyn Pangonis

Theppharit Panichsillapakit

V. Shane Pankratz

Marcus Panning

Jesse Papernburg

Georgios Pappas

Peter Pappas

Bharat Parekh

Luis Parra-Rodriguez

Michael Parry

Eshan Patel

Nimish Patel

Pratish Patel

Rupa Patel

Atul Patel

David Paterson

Preeti Pathela

Jayani Pathirana

Sarang Patil

Shelia Patrick

Jeffrey Pearson

Nicole Pecora

Laura C Pedraza-Arévalo

Giles Peek

Andrew Pekosz

Tuula Pelkonen

Laura Pellegrinelli

Stephen Pelton

Marianoel Pereira Gómez

Jose Perez-Molina

Olivier Pernet

Anna Person

Eskild Petersen

April Pettit

Varun Phadke

Patrick J. Phillips

Anne Piantadosi

Shiv Pillai

Liise-anne Pirofski

Jason Pogue

Christopher Polage

Nira Pollock

Corneliu Popescu

Stephen Popper

Stephanie Pouch

Virginie Prendki

D Rebecca Prevots

Andrea Prinzi

Bonnie Prokesch

Olha Puhach

Matthew Pullen

Shohra Qaderi

Firdausi Qadri

Sonja Raaum

Anastasia Rakow

Ruchir Raman

Meena Ramchandani

Nanda Ramchandar

Mayur Ramesh

Danielle Rankin

Mana Rao

Krishna Rao

Agam Rao

Tamar Ratishvili

Adriana Rauseo

Hariharan Regunath

Francoise Renaud

Marc Rendell

Hilary Reno

Harry Reyes Nieva

Nathaniel Rhodes

Matteo Ricco

Veronica Richards

Lauren Richey

Aaron Richterman

David Riedel

Patricia Riggs

Christina Rivera

Karla Rivera Rivera

Nesrine Rizk

Reuben Robbins

Scott Roberts

Joan Robinson

Jessica Robinson-Papp

Jaime Robledo

Joseph Rocco

Paula Rodas

Kyle Rodino

Jesus Rodriguez-Bano

Alfonso J. Rodriguez-Morales

Guillermo Rodriguez-Nava

Keith Rodvold

Ralph Rogers

Mary-Claire Roghmann

Marijo Roiko

Matthew Romo

Dusten Rose

Victor Rosenthal

Jennifer Ross

Geoffrey Rossi

Sarah Rowan

Christopher Rowley

Pavitra Roychoudhury

Marco Ruiz

Stefano Rusconi

Alessandro Russo

Olli Ruuskanen

Keenan Ryan

Michael Rybak

Jonathan Ryder

David Sack

Ryuichi Sada

Siddhartha Saha

Lisa Saiman

George Sakoulas

Carlos Saldana

Ezzeldin Saleh

Jon Salmanton-Garcia

Helene Salvator

Suryaprakash Sambhara

Vittorio Sambri

Indy Sandaradura

Joseph Sassine

Michael Satlin

Jennifer Saullo

Alexandra Savinkina

Lynora Saxinger

Henk D. F. H. Schallig

Dena Schanzer

Marc Scheetz

Sam Scheiner

Georgios Schinas

Amanda Schnee

Christof Manuel Schoenenberger

Asher Schranz

Audrey Schuetz

Rossana Scutari

Carlos Seas

Sharvan Sehrawat

Allan Seibert

Inna Sekirov

Noritaka Sekiya

Júlia Sellarès-Nadal

John Sellick

Parham Sendi

Edward Septimus

David Serota

Joe Sexton

Robert Shafer

Neel Shah

Shokoofeh Shamsi

G. Shanks

Adrienne Shapiro

Kareem Shehab

Samuel Shelburne

Jenny Shen

Jacqueline Sherbuk

Renslow Sherer

Ryan Shields

Nathan Shively

Dennis Shoemaker

Shmuel Shoham

Kensuke Shoji

Andrew Shorr

adrienne showler

Nabin Shrestha

Bhavarth Shukla

Jonathan Shuter

Thiago Silva

Richard Silvera

Gordana Simeunovic

Matthew Simon

Michelo Simuyandi

Sarman Singh

Inderpaul Singh

Edford Sinkala

Janos Sinko

Geetha Sivasubramanian

Gwenn Skar

Andrew Skinner

Zachary Smith

Luke Blagdon Snell

Jonathan Snowden

Graham Snyder

Brittney Snyder

David Snydman

Mahdee Sobhanie

Maria Soderlund-Venermo

Isaac Solomon

Mark Sonderup

Suneeta Soni

Vicente Soriano

Leonard Sowah

Anne Spaulding

Brad Spellberg

Jennifer Spicer

Owen Spiller

Matthew Spinelli

Roseanne Sprute

Suman Srinivasa

Kenneth Ssebambulidde

Samuel Stampfer

J. Erin Staples

Jack Stapleton

Ann Stapleton

Rick Starlin

Kim Steegen

Samantha Steiger

David Stein

Julie Steinbrink

Jeremy Steinbruck

Christoph Stephan

Kathryn Stephenson

Tyler Stone

Donald Storey

Katharine Stott

Jason Stout

Susan Stramer

Judy Streit

Robert Strengen Bigseth

Lara Strick

Amy Sturt

Chia-ping Su

Amanda Suchanek

Kimberly Sue

Nathan Summers

Kelsey Sumner

Maria Sundaram

Clara Suñer

Torgny Sunnerhagen

Bernard Surial

Jesse Sutton

Joji Suzuki

Talia H. Swartz

Lina S. Sy

Emily Sydnor Spivak

Rehan Syed

Julia Tabatabai

Bieke Tack

Don Bambino Geno Tai

Yuichiro Takeshita

Andrew Talal

Rohit Talwani

Pranita Tamma

Tina Tan

Darrell Tan

James Tang

Naoki Tani

Tomohiro Taniguchi

Janet Tate

Pierre Tattevin

Barbara Taylor

Stephanie Taylor

Pabio Tebas

Benjamin Teh

Elizabeth Temkin

Jaap ten Oever

Junko Terada-Hirashima

Jeffrey Tessier

Kinna Thakarar

Sanya Thomas

Mark Thomas

Dorothea Thompson

Melanie Thompson

George Thompson

Phyllis Tien

Dylan Tierney

L Gayani Tillekeratne

Tristan Timbrook

Jean-Francois Timsit

Amir Tirmizi

David Tobin

Ryan Tomlin

Steven Tong

Jessie Torgersen

Julian Torre-Cisneros

Jose Torres

Elizabeth Townsend

Takashi Toya

Hidenori Toyoda

Vianney Tricou

Trang Trinh

Julie Trivedi

Jason Trubiano

Shin-Yi Tsai

Nicole Ngai Yung Tsang

Raymond Tsang

Patrick Tschida

Alice Tseng

Yo Tsukamoto

Jean-Jacques Tudesq

Nicholas Turner

Frank Tverdek

Anne-Catrin Uhlemann

Netanya Utay

Hamid Vaez

Lucinda Van Anglen

Reinout Van Crevel

Thomas van der Vaart

Tjip van der Werf

David Van Duin

Cees van Nieuwkoop

Trevor Van Schooneveld

Maarten van Wijhe

Lilian Vargas Barahona

Jose Vazquez

Ana Beatriz Veiga

Alida Veloo

Arthur Vengesai

Francois Venter

Alessandra Vergori

Tarfa Verinumbe

Sten Vermund

Thomas Verstraeten

Timo Vesikari

Uffe Vest Schneider

Karen Vigil

Javier Villafuerte

Merceditas Villanueva

Nicole Vissichelli

Amy Vittor

Michael von Fricken

Jamie Wagner

Nicole Wagner

Gabriel Wagner

Noah Wald-Dickler

Lorne Walker

Harry Walker

Stephen Waller

Edward Walsh

Richard Wang

Jann-Yuan Wang

Jianing Wang

Celestine Wanjalla

Helen Ward

Adilia Warris

Laura Waters

R. Scott Watson

James Watson

Bethany Wattles

Victoria Weaver

Devin Weber

Melissa Weimer

Geoffrey A. Weinberg

Robert Weinstein

Scott Weisenberg

John Weiser

Maja Weisser

Elliott Welford

Chad Wells

William Werbel

Ryan Westergaard

Nils Wetzstein

Alice Wheeler

Jennifer Whitaker

Nicole White

Margaret White

Timothy Whitman

Katherine Wick

Andrew Wiese

Eli Wilber

Zanthia Wiley

Robert Wilkinson

Peter Williamson

John Williamson

Zachary Willis

John Wilson

Megan Wimmer

Alan Winston

Kevin Winthrop

Max Witt

Amy Wojciechowski

Joshua Wolf

Cameron Wolfe

Roman Wölfel

Joshua Wong

Junda Woo

Charles Woods

Darcy Wooten

Marylee Worley

Gary Wormser

Marjan Wouthuyzen-Bakker

William Wright

Cameron Wright

Henry Wu

Minkey Wungwattana

Alysse Wurcel

David Wyles

Anne Wyllie

Lixin Xie

Hang Xie

Yi Xu

Tetsuya Yagi

Kristina Yamkovoy

Katherine Yang

Trent Yarwood

Mohamad Yasmin

Daniel Yeoh

Zachary Yetmar

Leah Yoke

Jo-Anne Young

Yinong Young-Xu

Dingle Yu

Ki Wook Yun

Ahmed Zaiem

Danniel Zamora

Lingchan Zeng

Jerry S. Zifodya

Shanta Zimmer

Beatrice Zovich

Lina Zuccatosta

Jason Zucker

Julio Zuniga

Joseph R Zunt

